# Analgesia effect of lentivirus-siSCN9A infected neurons in vincristine induced neuropathic pain rats

**DOI:** 10.1080/21655979.2021.2008696

**Published:** 2021-12-18

**Authors:** Baojun Fu, Rong Zhu

**Affiliations:** a Department of Anesthesiology, The Sixth Affiliated Hospital of Guangzhou Medical University Qingyuan People Hospital, Qingyuan, Guangdong, China; b Department of Anesthesiology, The Second Xiangya Hospital,Central South University, Changsha, Hunan, China

**Keywords:** LV-siSCN9A infection, neurons, vincristine, neuropathic pain

## Abstract

At present, the mechanism of siSCN9A in Vincristine (VCR)-induced neuropathic pain (NP) is still unclear. This study aimed to explore the analgesic mechanism of lentivirus-siSCN9A (LV-siSCN9A) infected neurons against NP. 40 male Sprague-Dawley (SD) rats were divided into a control group (injected with normal saline), a model group (VCR-induced NP model), a LV-SC group (NP model mice were injected with LV-SC-infected dorsal root ganglia (DRG) neuron cells under the microscope), and a LV-siSCN9A group (NP model mice were injected with LV-siSCN9A-infected DRG neuron cells under the microscope, with 10 rats in each group. The changes of mechanical withdrawal threshold (MWT) and thermal withdrawal latency (TWL) of rats in different groups were detected by behavior testing, the Nav1.7 changes in each group were detected by immunofluorescence double standard and Western-blot method. It was found that compared with the control group, the MWT and TWL of the rats in model group were significantly decreased (*P* < 0.05), and the expression levels of Nav1.7 messenger ribonucleic acid (mRNA) and proteins were significantly increased (*P* < 0.05). Compared with LV-SC group, the MWT and TWL of rats in LV-siSCN9A group were significantly increased (*P* < 0.05), the expression levels of Nav1.7 mRNA and proteins were significantly decreased (*P* < 0.05), and the CGRP expression of spinal dorsal horn was significantly decreased. It was concluded that the LV-siSCN9A infected neurons could play an analgesic role by down-regulating Nav1.7 expression induced by VCR in NP model.

## Experimental design

**Figure:**
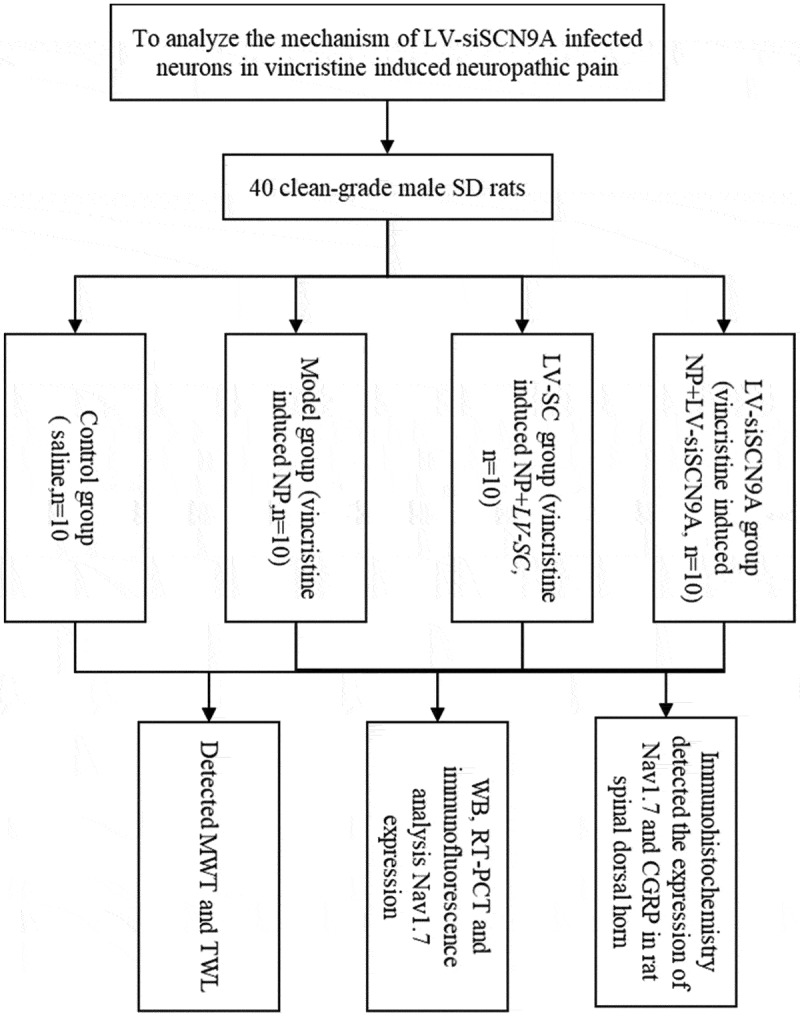

## 1. Introduction

The chemotherapeutic drug vincristine (VCR) has dose-dependent peripheral neurotoxicity, which can cause nerve injury and neuropathic pain (NP). The duration of NP is long and repeated attacks, and the treatment effect is poor, which seriously reduces the quality of life of cancer patients and limits the clinical application of chemotherapeutic drugs [1]. At present, the mechanism of NP is not fully understood, and most scholars believe that the pathogenesis includes peripheral and central mechanisms [2–4]. The peripheral mechanism includes the interactive conduction induced discharge of neurons, the excitability enhancement of adjacent undamaged fiber, and the abnormal discharge of damaged peripheral afferent fiber. The central mechanism includes the activation of the downstream facilitation system, the down-regulation of central inhibitory interneuron function, and the activation of neurons in the dorsal horn of the damaged spinal cord [5–8]. Studies have pointed out that in the NP animal model induced by VCR, the glial cells in the periaqueduct gray matter and spinal cord gray matter of the midbrain of rats are significantly activated after administration, which may be related to the up-regulation of the inflammatory factor interleukin (IL)-1β released by glial cells and the down-regulation of the expression of glial cell-derived neurotrophic factor in the spinal dorsal horn [9]. After spinal cord peripheral nerve injury, microglia in the spinal cord can be activated. Activated microglia is a key cell mediator in the pathogenesis of pain and hypersensitivity caused by nerve injury, suggesting that central sensitization is involved in the production of NP [10]. There are a variety of protein expression differences in the cerebral cortex tissues of VCR-induced NP. The differentially expressed proteins include DJ-1, heat shock protein 10, Cu-Zn superoxide dismutase, and adenosine triphosphate (ATP) synthase D, indicating that VCR-induced NP may have a central regulatory mechanism [11].

The conduction and regulation of pain signals depend on the activity of ion channels on afferent fibers, in which voltage-gated channels and receptor-gated channels jointly regulate resting membrane potential and action potential [12]. It has been verified that some channel subtype genes may be used as potential drugs for the treatment of chronic pain in humans [13]. The sodium ion channels as the basis of excitatory cell resting potentials and action potentials are considered as potential research targets for analgesic therapy [14]. Nav1.3, Nav1.7, Nav1.8, and Nav1.9 in sodium ion channels are closely related to dorsal root ganglia (DRG) neuron damage [15]. Recently, studies have shown that voltage-gated sodium ion channels IKα subunits (Nav1.7, SCN9A coding) may play an important role in abnormal pain disorders [16–19]. A previous studies showed that Nav1.7 nonsense mutations can trigger congenital painless disease, while Nav1.7 dysfunction mutations can trigger paroxysmal severe pain and limb red pain [20]; 21). Voltage-gated sodium ion channels Nav1.7 were mainly expressed in DRG and sympathetic ganglia [22–24]. A variety of sodium and potassium ion channels are expressed in the DRG, and these ion channels have the function of regulating the transport of synapses as well as the conversion and conduction of nerve signals [25]. After nerve injury, the undamaged or damaged neurons will have abnormal discharge activity [26–28]. It is speculated that this abnormal put point of neurons may be closely related to the abnormal expression of Nav1.7.

Based on the current research results, LV-siSCN9A plays an important role in VCR-induced NP, but the specific role and mechanism of action are currently unclear. In this study, a LV-mediated siSCN9A was constructed to infect neurons and act on VCR-induced NP in rats to explore the effect of LV-siSCN9A-infected neurons on VCR-induced NP in rats and analyze the specific mechanism of LV-siSCN9A-infected neurons in NP, aiming to provide an important theoretical and experimental basis for transgenic therapy of NP.

## 2. Materials and method

### 2.1. Laboratory animals and groups

40 clean-grade male SD rats were 10 weeks old and weighed about 200 g. All animals were fed in national standard rodent feed cages, 4 in each cage, free diet, and no significant difference in weight between groups. All animals were raised in 12 h light and dark alternate environment and free diet. The room was regularly disinfected, temperature was controlled at 20–26°C and humidity was controlled in 40–50%. All of them were adaptively fed for 2 weeks. Animal handling and experimental procedures were in accordance with the national experimental animal norms and the ethics committee had approved.

Rats were randomly divided into 4 groups: a control group, a model group, a LV-SC group, and a LV-siSCN9A group, with 10 rats in each group.

### 2.2. Establishment of a rat model of vincristine induced NP

From the date of the experiment in the control group, the abdominal cavity of rats was injected with saline 1 mL in odd day and the stomach of rats was irrigated with saline 1 mL in even day. A rat model of VCR-induced NP was established by referring to the method of 29. In model group, LV-SC group and LV-siSCN9A group, the abdominal cavity of rats was injected with VCR (Haizheng Pfizer Pharmaceutical, Co. Ltd) 100 μg/(kg·d) in odd day and the stomach of rats was irrigated with saline 1 mL in even day. Every group was given the drug continuously for 16 days.

### 2.3. LV-SC and LV-siSCN9A injection in rats under artificial intelligence-based digital microscope

Operating the 3D micromanipulator knob and rotating the fine-tuning section under an artificial intelligence-based digital microscope allowed the glass microelectrode tip to enter the DRG positive middle position vertically. A slight depression appeared in the DRG under the microscope, and the anterior and posterior diameters became shorter. The flow rate of the microinjection pump was set for 12 μL/h, microinjection was turned on, and the tip of the electrode was fine-tuned and made it back. The depression of the DRG gradually recovered until the injection material (virus and the mixture of its dyes, LV-scramble, LV-siSCN9A) was completely diffused in the DRG. The injection material was uniformly diffused within the DRG at a uniform rate of 10 min, and the DRG had a full appearance. After stopping the infusion, wait for 10 min so that the injected substance was fully diffused.

### 2.4. Changes of mechanical withdrawal threshold (MWT) and thermal withdrawal latency [TWL) in rats detected by behavior testing

The changes of MWT and TWL in different groups of rats were detected using the method introduced by 30, which was modified slightly in this study. MWT values of rats in each group were measured 1 day before and 4, 8, 16 days after modeling, and the time of each measurement was chosen from 7:00a.m. to 8:00a.m. Indoor was kept quiet and the room temperature was controlled at 22 ± 2°C. The MWT was determined by Von Frey filament pain detector (USA Stoeiting]. Six von Frey hair, with logarithmically increasing in strength, were selected and the bending forces measured were 1, 2, 4, 6, 8, and 15 g, respectively. The rats in each group were placed on the metal grid and covered with the transparent glass observation frame. First, the rats were placed on the grid for 20 minutes so that they can adapt to the environment, and the measurement was started from 2 g. The cilia were vertically pricked to the lateral 1/3 of the posterior plantar skin of the rats through the mesh, and then them were bent into an S shape with slight force. Each stimulation lasted for 10s, avoiding the foot pad. The interval between two stimuli was 10–15s, to observe whether rats had rapid foot contraction reflex or foot licking response during stimulation time or when the von Frey needle was removed. If there was a response, it was recorded as positive, and if there was no response, it was recorded as negative. The adjacent decreasing von Frey hair was selected for stimulation if the foot constriction response was positive; the adjacent increasing von Frey hair was selected for stimulation if it is negative. Stimulation could be stopped when the following situations occurred: the cumulative measurement reached 9 times; the response was still negative when the intensity of the stimulus reached 15 g; after the first positive response, it was continuously measured 4 times. After the test, and the foot response mode (g) was converted to the corresponding foot retraction threshold (N) by a specific program. It was regarded as abnormal pain sensitivity if the 50% MWT value of the rats left posterior foot was ≤9 g, and the rats were removed.

### 2.5. The Expression of Nav1.7 Cell Types on DRG Neurons Observed by Immunofluorescence Double Standard Method

On the 7^th^ day after DRG injection, rats in LV-SC group and LV-siSCN9A group were put to death under anesthesia. The spinal cord, left and right sides DRG specimens were taken and immediately put into normal saline. The spinal cord and DRG were cut at the nerve root, section was faced down and placed in a section box to make frozen sections. The freezer chamber was continuously temperature controlled at −20°C, fixed with an optimal setting temperature embedding agent, and sliced with 5 μm thickness after 10 min. The sections were cut and observed within 12 h by fluorescence microscope (Leica, Germany) under an argon ion laser with internal excitation wavelength of 488 nm.

### 2.6. The Expression Level of Nav1.7 In Each Group Tested by Western-blot Method

The L_4-5_ DRG was placed in an electric tissue homogenizer (Kimble, USA). The cell lysate and benzyl sulfonyl fluoride (Beijing Kariky Biotechnology, Co. Ltd., China) were added at a ratio of 1:1000 and then homogenized. The tissue was placed on ice for 30 min to fully crack. After centrifugation (12,000 r/min) in a precooled centrifuge for 15 min, the supernatant was transferred to a precooled Eppendorf tube and the total protein concentration was detected in strict accordance with the instructions of the Bicinchonic acid (BCA) protein concentration assay kit (Thermo Fisher scientific, USA). 4× buffer protein loading buffer was added at a ratio of 1:3 and boiled 10 min at 100°C to make protein denaturation. The tissue was stored at −80°C after cooling at room temperature. The protein supernatant was absorbed for 10 μL, and the sample was added according to the instructions of the BCA kit. The absorbance of each protein was detected by the automatic enzyme labeling instrument (Molecular Devices, USA). The standard curve was drawn from the absorbance of the working solution, and the protein concentration of the sample was calculated.

### 2.7 The expression of Nav1.7 detected by quantitative PCR

First, Ribonucleic acid (RNA) was extracted from the spinal cord of rats in each group. In the collected tissues, 1 mL of Trizol was added. The tissue was frozen and thawed three times between liquid nitrogen and 37°C water bath, and whirled for 30s. After centrifugation at 40°C for 10 min, the clarified Trizol products were absorbed into a new centrifuge tube. After 5 min at room temperature, the sample was fully cracked. 0.2 ml chloroform was added to each milliliter of Trizol, mixed with vortex for 15s, and placed at room temperature for 3 min. 12,000 g sample was centrifuged at 40°C for 15 min. Then, the upper colorless aqueous phase containing total RNA was taken into a new centrifuge tube, 0.4 mL of Trizol was taken from per mL, and 0.4 mL isopropanol was added. Then, it was necessary to reverse for several times to mix well, and make it precipitate at room temperature for 10 min. After 12,000 g sample was centrifuged at 40°C for 10 min, RNA precipitated at the bottom of the tube and the supernatant was discarded. The concentration and purity of RNA were determined. Then, reverse transcription was needed. In EP tube, 1 μg total RNA was extracted and complementary DNA (cDNA) was synthesized by reverse transcription-polymerase chain reaction (RT-PCR) Kit (TaKaRa, China). quantitative RT-PCR (qRT-PCR) amplification was performed. The internal reference was glyceraldehyde-3-phosphate dehydrogenase (GAPDH) (China Shenggong Biotechnology Co., Ltd.). The reaction system was 10 μL, and the reaction conditions were pre denaturation at 95°C for 30s, denaturation at 95°C for 5s, annealing at 60°C for 20s and elongation at 72°C for 30s. A total of 40 cycles were performed. The Ct value was recorded and the expression of related genes was calculated.

### 2.8. The Expression of Nav1.7 And Calcitonin Gene Related Peptide (CGRP) in Rat Spinal Dorsal Horn Detected by Immunohistochemistry Method

The rats were anesthetized with an intraperitoneal injection of 10% chloral hydrate (Shanghai Shifeng Biotechnology, Co. Ltd., China) at a dose of 3.5 mg/kg, and 500 mL warm PBS was quickly irrigated into the rats through the right ventricle of the heart. After washing the blood, the rats were fixed with 4% paraformaldehyde (Nanjing Huaxi Chemical, Co. Ltd., China). After the left L5 DRG and lumbar swelling section of the spinal cord were fixed overnight, gradient alcohol was dehydrated, conventional paraffin embedding was performed, and sections were made. Three complete sections of lumbar swelling were taken from each specimen, which dewaxed to water, washed with PBS for 3 × 5 min, and putted into 3% hydrogen peroxide (H_2_O_2_) at 37°C for 20 min. Then these sections were washed with PBS for 3 × 5 min, washed with PBS for 3 × 5 min after antigen repair, and dropped with 3% goat serum blocking solution (Anhui Jingke Biotechnology, Co. Ltd., China). After 20 min, the primary antibody was diluted according to the following ratio: mouse monoclonal antibody against Nav1.7 (1:600), horseradish peroxidase-labeled goat against mouse (1:1000), rabbit against monoclonal antibody CGRP (1:1000). The negative control was replaced with PBS, placed at 37°C for 1 h, and overnight at 4°C. After reheating at 37°C, PBS was used to wash for 3 × 5 min. The diluted solution of 50 μL secondary antibody was added, placed at room temperature for 20 min, and washed with PBS for 3 × 5 min. 4ʹ6-diamidino-2-phenylindole (Beijing Kariky Biotechnology, Co. Ltd., China) (1:200) was added to dye the kernels for 1 min and washed with PBS for 3 × 5 min. The sections were sealed with anti-fluorescence attenuation sealant, observed under a fluorescence microscope and preserved by taking pictures.

### 2.9. Statistical analysis

Statistical analysis was performed using SPSS26.0 software. All data were expressed as mean ± standard deviation ($\bar{X}$ ± s). Comparisons between groups and within groups were performed by analysis of variance, and *P* < 0.05 was considered as statistical significance. After comparison on means of multiple groups through the *F*-test, further pairwise comparisons between the means (also called multiple comparisons) were made if a statistically significant conclusion was reached. The Student's *t*-test (LSD-t) was adopted to confirm the experimental research. At the design stage, the pairwise comparisons between some means were determined according to the research objective or professional knowledge. The counting data was expressed as incidence n (%), χ^2^ was used to test comparison, and *P*< 0.05 considered the difference to be significant.

## 3. Result

### 3.1. Results of body weight and paw thickness of rats in each group

LV-siSCN9A may have a certain analgesic effect in VCR-induced NP, and this analgesic effect may be related to the changes in the expression of the Nav1.7 protein encoded by the SCN9A gene. Therefore, based on the rat model of VCR-induced NP, the specific mechanism of LV-siSCN9A in VCR-induced NP and whether it was related to the expression of Nav1.7 gene and protein was analyzed in this study.

**Figure 1.**
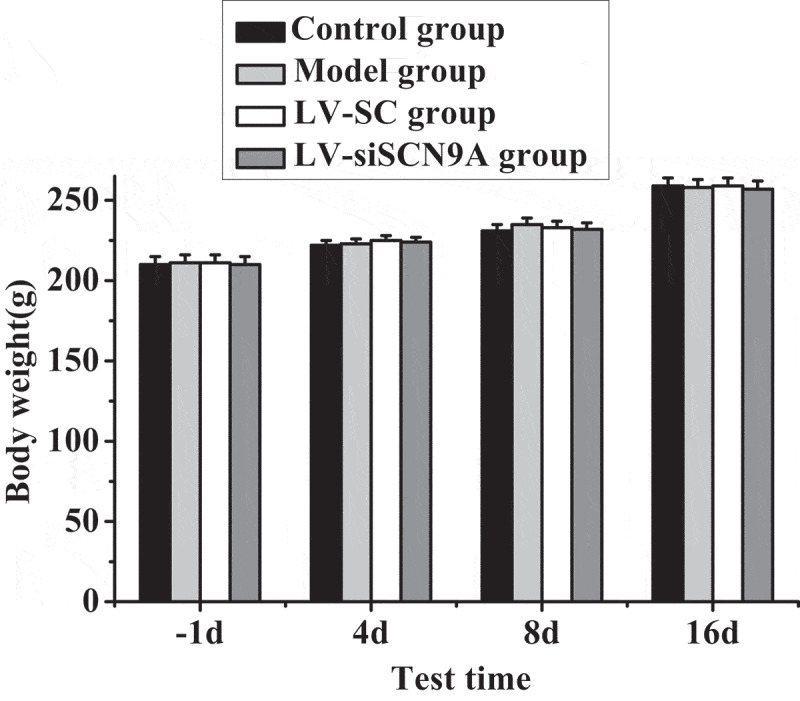
Body weight test results of rats in each group (a: compared with the control group, P < 0.05; b: compared with LV-SC group, P < 0.05)

**Figure 2.**
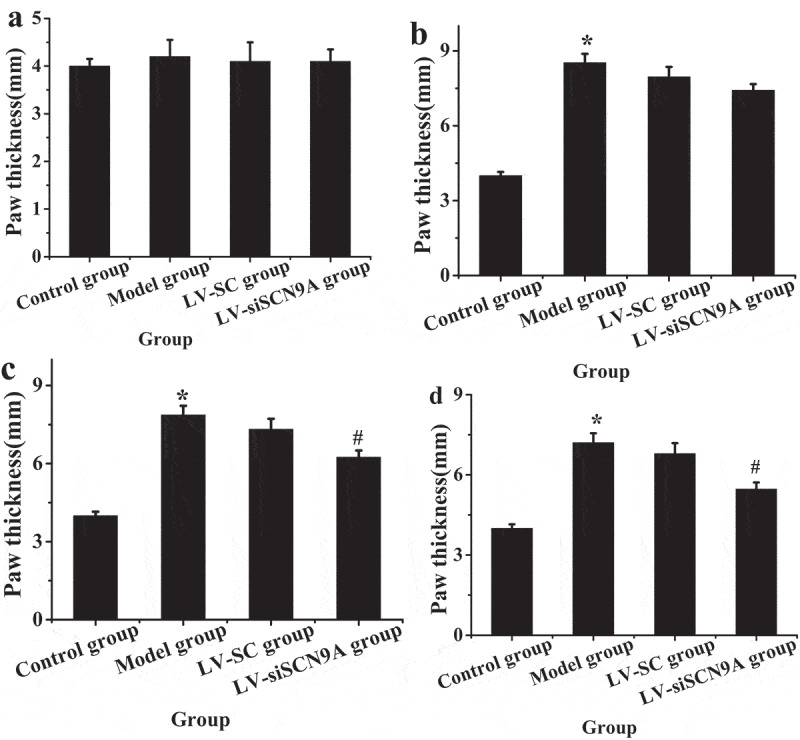
Detection results of paw thickness of rats in different groups before and after modeling (A. 1 day before modeling; B. 4 days after modeling; C. 8 days after modeling; D. 16 days after modeling; (* meant *P* < 0.05 compared with the control group; # suggested *P* < 0.05 compared with LV-SC group)

Figures 1 and 2 show the body weight and the paw thickness of the rats in each group. By detecting the weight of rats before and after modeling, it was found that the weight of rats in each group increased gradually with time. However, there was no significant difference in body weight among the groups (P > 0.05). The paw thickness of the rats in different groups was measured before and after modeling. The results showed that there was no significant difference in the paw thickness of rats in each group 1 day before modeling. The paw thickness of rats in model group, LV-SC group and LV-siSCN9A group increased significantly 4 days after modeling, with statistical difference compared with the control group (P < 0.05). With the increase of modeling days, compared with the control group, the paw thickness of rats in model group, LV-SC group and LV-siSCN9A group showed a downward trend. Compared with the control group, the paw thickness of rats in the model group still showed a significant increasing trend from 8 days to 16 days after modeling. Compared with the model group, the paw thickness of rats in LV-siSCN9A group decreased significantly (P < 0.05).

### 3.2. Test results of MWT and TWL in each group of rats

**Figure 3.**
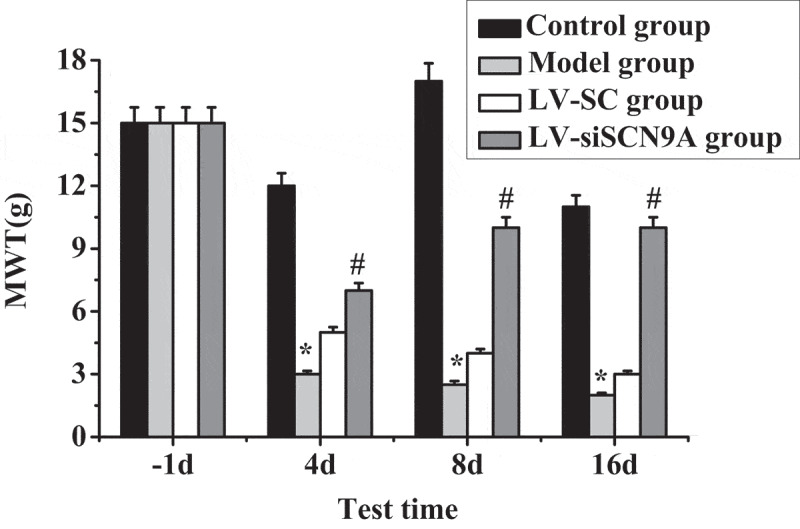
Test results of MWT (* meant *P* < 0.05 compared with the control group; # suggested *P* < 0.05 compared with LV-SC group)

**Figure 4.**
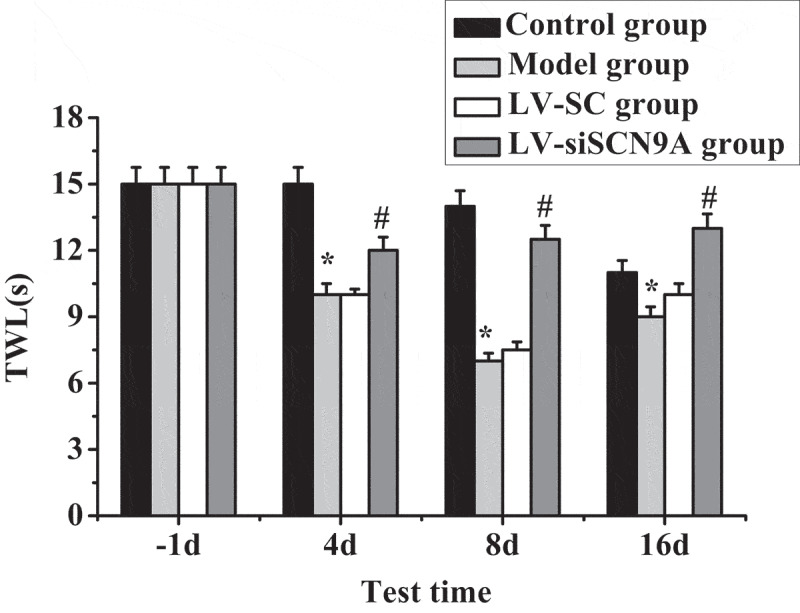
Test results of TWL (* meant *P* < 0.05 compared with the control group; # suggested *P* < 0.05 compared with LV-SC group)

Test results of MWT and TWL in each group of rats are shown in Figures 3 and 4. It was shown that the MWT and TWL of the model group rats were decreased after modeling. Compared with the control group, the MWT and TWL of each detection time in the model group were significantly decreased after modeling, and the difference was statistically significant (P < 0.05). Compared with the LV-SC group, the MWT and TWL of each detection time in the LV-siSCN9A group were significantly increased after modeling, and the difference was statistically significant (P < 0.05).

### 3.3. Observation Results of Immunofluorescence Double Standard Method

**Figure 5.**
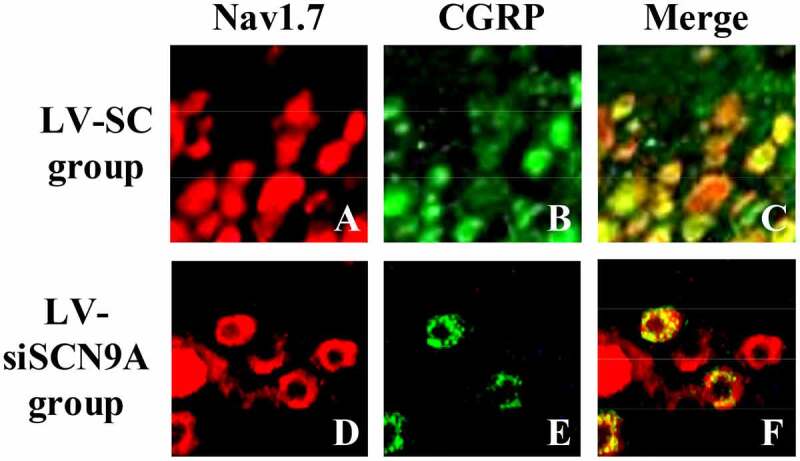
Observation results of immunofluorescence double standard method (A: expression of Nav1.7 in LV-SC group; B: CGRP positive cells in LV-SC group; C: double staining map of LV-SC group; D: expression of Nav1.7 in LV-siSCN9A group; E: CGRP positive cells in LV-siSCN9A group; F: double staining map of LV-siSCN9A group)

**Figure 6.**
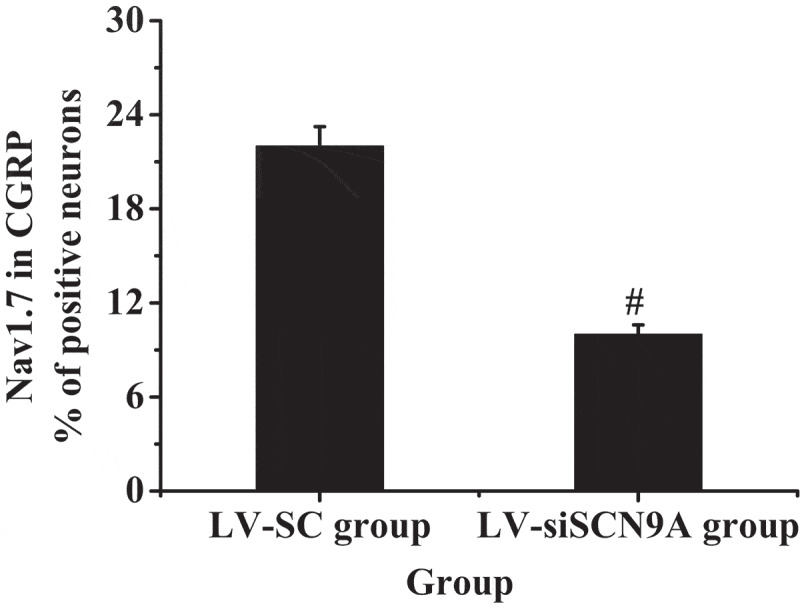
The percentage of Nav1.7 expressed in CGRP positive cells (# suggested *P* < 0.05 compared with LV-SC group)

The results of immunofluorescence double standard method were shown in Figures 5 and 6. It was shown that the expression of Nav1.7 was mainly on CGRP positive cells. The expression of Nav1.7 on CGRP positive cells in LV-siSCN9A group was significantly decreased compared with LV-SC group, and the difference was statistically significant (P < 0.05).

### 3.4. Expression of related proteins in rats with pathological pain

**Figure 7.**
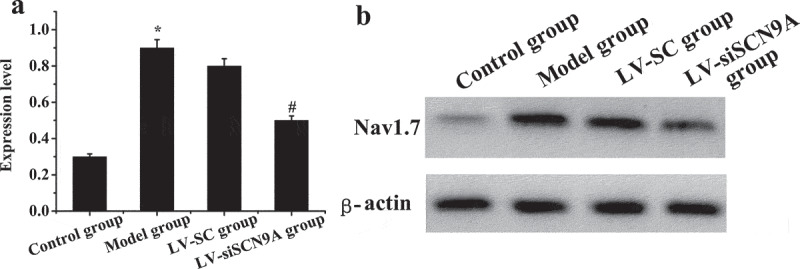
The test results of Nav1.7 Western-blot method (A: Nav1.7 protein expression of rats in each group; * meant *P* < 0.05 compared with the control group; # suggested *P* < 0.05 compared with LV-SC group; B: Western blotting)

**Figure 8.**
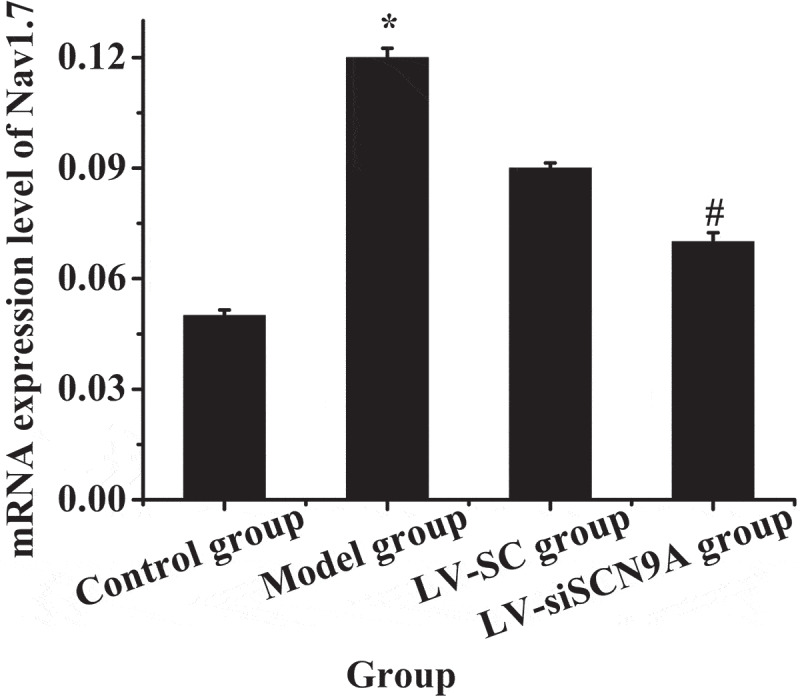
The mRNA expression level of Nav1.7 in each group (* meant *P* < 0.05 compared with the control group; # suggested *P* < 0.05 compared with LV-SC group)

The expression levels of Nav1.7 mRNA and protein in each group were analyzed. The results were shown in Figures 7 and 8. Compared with the control group, the expression level of Nav1.7 mRNA and proteins in the rats lumbar swelling of spinal cord was significantly increased in the model group. Compared with LV-SC group, the Nav1.7 mRNA and protein expression in LV-siSCN9A group was significantly decreased, and the difference was statistically significant (P < 0.05).

**Figure 9.**
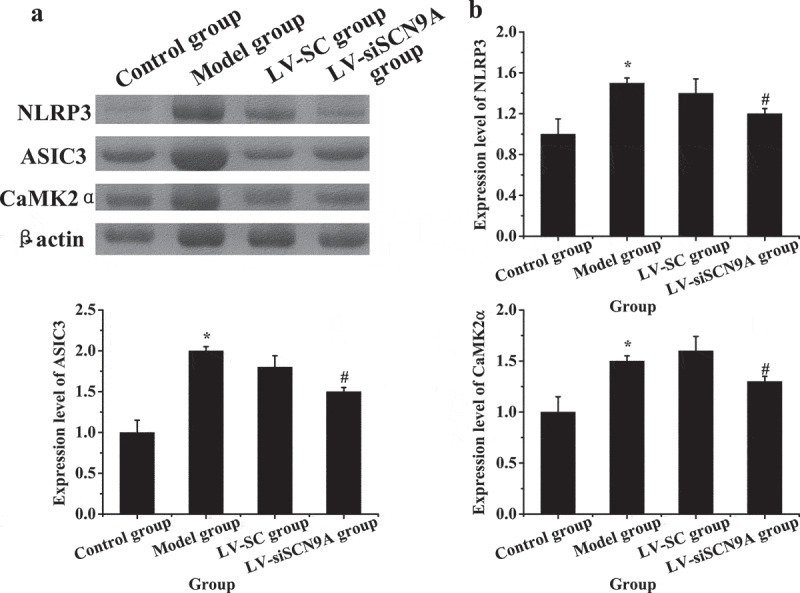
Expression of related proteins in rats with vincristine induced pathological pain (A: Western blot electrophoresis; B. NLRP3 protein; C. ASIC3 protein; D. CaMK2α protein; * meant *P* < 0.05 compared with the control group; # suggested *P* < 0.05 compared with LV-SC group. B: Western blotting)

As shown in Figure 9, compared with the control group, the expressions of NLRP3, ASIC3 and CaMK2α in the spinal cord of the model group were significantly increased (P < 0.05), and compared with the model group, the expressions of NLRP3, ASIC3, and CaMK2α in the spinal cord of the LV-siSCN9A group were significantly decreased (P < 0.05). The expression levels of NLRP3, ASIC3 and LV-siSCN9A in the spinal cord of LV-SC group had no significant change (P > 0.05).

### 3.4. Test results of immunohistochemistry

**Figure 10.**
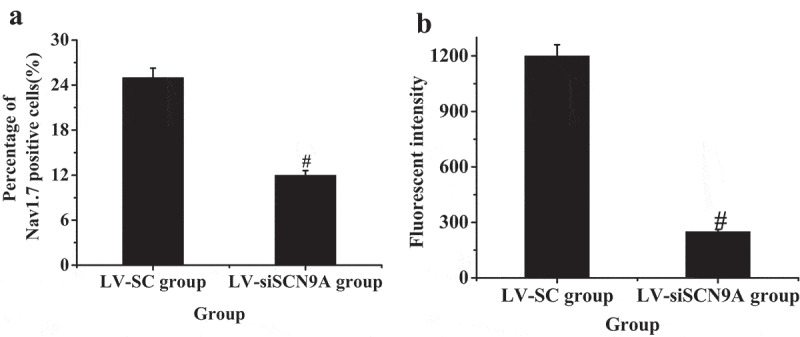
Test results of immunohistochemistry (A: percentage of Nav1.7 positive cells; B: CGRP fluorescence intensity; (# suggested *P* < 0.05 compared with LV-SC group)

The percentage of Nav1.7 positive cells in the LV-siSCN9A group was significantly decreased (P < 0.05) compared with the LV-SC group, and the difference was statistically significant (0.05). The results were consistent with Western-blot. The CGRP expression of spinal dorsal horn in LV-siSCN9A group was significantly decreased compared with that in the LV-SC group, and the difference was statistically significant (P < 0.05) (Figure 10).

## 4. Discussion

The content of CGRP in spinal dorsal horn is the highest in the nervous system of human and rat [31,32]. In addition, the content of DRG, CGRP in trigeminal and pituitary is also higher. Studies have shown that CGRP is closely associated with NP [33–35]. The regulation of CGRP in vivo is relatively complex, and it has many kinds of active substances associated with NP [36,37]. Therefore, the mechanism of CGRP in NP has been fully understood. The secondary effects of DRG and a large amount of CGRP released in the spinal dorsal horn play an important role in the transmission and regulation of pain information [38]. The male SD rats are taken as the research object to establish the rat model of VCR-induced NP, LV-SC, and LV-siSCN9A are injected into the rats under artificial intelligence-based digital microscope, and the changes of MWT and TWL are detected by behavior testing. The results show that compared with the control group, the MWT and TWL of each detection time in the model group are significantly decreased after modeling, and the difference is statistically significant (P < 0.05). Compared with the LV-SC group, the MWT and TWL of each detection time in the LV-siSCN9A group are significantly increased after modeling, and the difference is statistically significant (P < 0.05). It shows that LV-siSCN9A infected neurons can produce analgesic effect on VCR-induced NP rats.

DRG plays an extremely important role in neuroprotection, nerve injury, repair and extension of nerve axons in vitro, and the influence of noxious stimuli such as heat and pain on ion channels and other related biological experiments and pharmacological research [39]. In DRG neurons, voltage-gated sodium channels play an important role in the occurrence of pain. Rat DRG expresses tetrodotoxin-sensitive sodium channels (Nav1.1, Nav1.2, Nva1.6, and Nav1.7) [40]. Among them, the Nav1.7 channel may play a major role in the generation of ectopic discharge [41]. SCN9A was located on chromosome 2. The voltage-gated sodium channel Nav1.7, which encoded tetrodotoxin-sensitive, was overexpressed in sympathetic ganglia and peripheral neurons. SCN9A gene variation could cause pain-related diseases [42,43]. Recently, more and more evidence has shown that mutations in the SCN9A gene are closely related to abnormal pain. Among them, paroxysmal severe pain and primary erythematous limb pain are all due to misintentional mutations in certain positions of the human SCN9A gene sequence encoding Nav1.7 protein [44,45]. DRG neuron Nav1.7 has a high expression in the inflammatory pain model of rats 46), and the SCN9A gene mutation rat of DRG neuron has significantly reduced responsiveness to inflammatory pain [47,48]. The cell types Nav1.7 expressed on DRG neurons were further observed by immunofluorescence double standard method. The mRNA and protein expression levels of Nav1.7 in each group was detected by Western-blot method and quantitative PCR, and the expression of CGRP in the dorsal horn of rat spinal cord was detected by immunofluorescence. It was found that compared with the control group, the Nav1.7 mRNA and protein expression level of rats in the model group was significantly increased (P < 0.05). Compared with LV-SC group, the Nav1.7 mRNA and protein expression level of LV-siSCN9A group was significantly decreased [P < 0.05), and the CGRP expression of spinal dorsal horn was significantly decreased. It indicated that the high expression of Nav1.7 was related to peripheral nerve injury. The neurons infected by LV-siSCN9A could inhibit the expression of Nav1.7, and inhibit the expression of NLRP3, ASIC3 and CaMK2α in the spinal cord of rats with pathological pain, so as to achieve the effect of reducing NP in rats. The experiment was in line with expectations. 48,showed that SCN9A was closely related to pain, and its encoded Na^+^ channel protein Nav1.7 could amplify the initial pain signal and promote continuous nerve excitation (Jung et al., 23 × 17]. It was consistent with the results of the experiment.

Although the microinjection under the direct vision of DRG increases the difficulty of the operation, only by successfully mastering the details of the anatomy of the dorsal root ganglion can it be possible to directly inject si RNA successfully. For gene therapy, DRG local injection avoids the adverse effects caused by systemic application. Direct transfection of siRNA-SCN9A into neuronal cells can down-regulate the expression of Nav1.7, but si RNA is unstable and easily degraded. Therefore, in this study, LV-siSCN9A can effectively reduce the degradability of siSCN9A and achieve the effect of alleviating neuropathic pain, which provided a reliable theoretical basis for the clinical research of transgenic treatment of NP.

To sum up, NP is relieved by exploring the analgesic effect of LV-siSCN9A infected neurons on VCR-induced NP rats, and LV-siSCN9A infected neurons can effectively down-regulate the high expression of Nav1.7, which has important guiding significance for the treatment of NP in clinic.

## 5. Conclusion

The analgesic effect of LV-siSCN9A infected neurons in rats with NP induced by vincristine is in male SD rats. It was found that LV-siSCN9A infected neurons were injected into DRG can inhibit the Navl.7 high expression caused by peripheral nerve injury and relieve NP. This study provided experimental basis for transgenic analgesia and showed important theoretical significance. But there were also some shortcomings in the research process. For example, the small amount of data collection of samples led to a certain degree of deviation of the results. Therefore, the data capacity had to be further increased in the later research process, which made the results obtained more reference value.

## Highlights

- LV-siSCN9A infected neurons can aggravate the MWT and TWL of VCR-induced NP.
- LV-siSCN9A infected neurons can reduce the expression of NAV1.7 and Calcitonin Gene Related Protein (CGRP) in DRG in rats with neuropathic pain induced by vincristine.
- LV-siSCN9A infected neurons exert analgesic effect by regulating the expression of Nav1.7 in the blood vessel.
